# *En bloc* preparation of *Drosophila* brains enables high-throughput FIB-SEM connectomics

**DOI:** 10.3389/fncir.2022.917251

**Published:** 2022-12-16

**Authors:** Zhiyuan Lu, C. Shan Xu, Kenneth J. Hayworth, Song Pang, Kazunori Shinomiya, Stephen M. Plaza, Louis K. Scheffer, Gerald M. Rubin, Harald F. Hess, Patricia K. Rivlin, Ian A. Meinertzhagen

**Affiliations:** ^1^Department of Psychology and Neuroscience, Life Sciences Centre, Dalhousie University, Halifax, NS, Canada; ^2^Janelia Research Campus, Howard Hughes Medical Institute, Ashburn, VA, United States; ^3^Department of Cellular and Molecular Physiology, Yale School of Medicine, New Haven, CT, United States; ^4^Yale School of Medicine, New Haven, CT, United States; ^5^Applied Physics Laboratory, Johns Hopkins University, Laurel, MD, United States

**Keywords:** *Drosophila*, brain, fixation, connectomics, FIB-SEM, PLT, electron microscopy, sample preparation

## Abstract

Deriving the detailed synaptic connections of an entire nervous system is the unrealized goal of the nascent field of connectomics. For the fruit fly *Drosophila*, in particular, we need to dissect the brain, connectives, and ventral nerve cord as a single continuous unit, fix and stain it, and undertake automated segmentation of neuron membranes. To achieve this, we designed a protocol using progressive lowering of temperature dehydration (PLT), a technique routinely used to preserve cellular structure and antigenicity. We combined PLT with low temperature *en bloc* staining (LTS) and recover fixed neurons as round profiles with darkly stained synapses, suitable for machine segmentation and automatic synapse detection. Here we report three different PLT-LTS methods designed to meet the requirements for FIB-SEM imaging of the *Drosophila* brain. These requirements include: good preservation of ultrastructural detail, high level of *en bloc* staining, artifact-free microdissection, and smooth hot-knife cutting to reduce the brain to dimensions suited to FIB-SEM. In addition to PLT-LTS, we designed a jig to microdissect and pre-fix the fly’s delicate brain and central nervous system. Collectively these methods optimize morphological preservation, allow us to image the brain usually at 8 nm per voxel, and simultaneously speed the formerly slow rate of FIB-SEM imaging.

## Introduction

**FIGURE 1:**
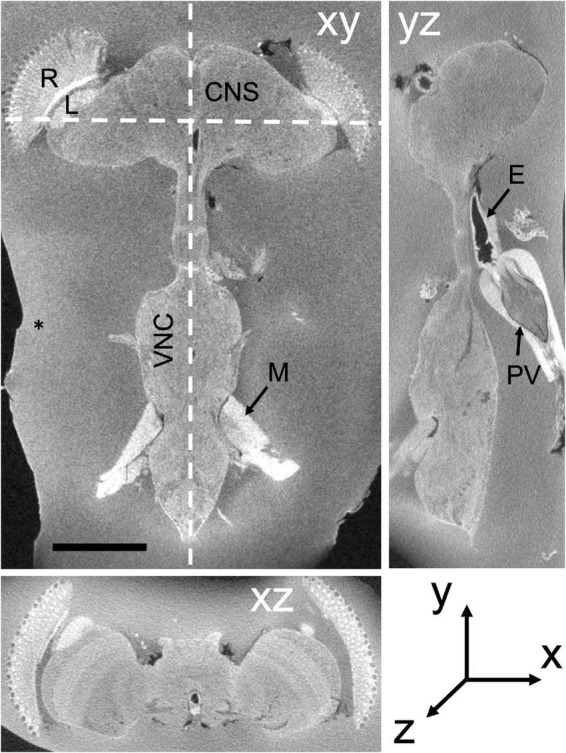
Soft X-ray dissected *Drosophila* CNS in three orthogonal planes, xy, yz, and xz. xy: frontal slice of the entire CNS, including the VNC. yz sagittal plane. xz transverse plane. Different zones in the surrounding BSA reveal successive additions of the cross-linked protein. E, esophagus; L, lamina; M, muscle; R, retina; PV, proventriculus. Scale bar in xy: 200 μm.

Increasingly rapid progress is being made to secure the exact synaptic wiring diagram of a brain, its connectome (Lichtman and Sanes, 2008), complete at the electron microscope (EM) level. That knowledge will enable functional analyses of synaptic circuits, and so help reveal the mechanism of identified behaviors. Attention is directed mostly to the model brains of genetically manipulable species (Luo et al., 2008), especially those of the mouse and the fruit fly *Drosophila melanogaster* (Figure 1). The *Drosophila* brain contains ∼100,000 neurons (Shimada et al., 2005; Meinertzhagen, 2018), 1,000 times fewer than in the mouse; this has enabled significant progress on the fly, despite the wide range of methods available for brains of other sizes (e.g., Hayworth et al., 2014; Kubota et al., 2018). However, *Drosophila*’s brain presents a special problem because even though the z-axis resolution for serial-section EM (ssEM) may be satisfactory for mouse brains (Denk and Horstmann, 2004; Hayworth et al., 2014; MICrONS Consortium, Bae et al., 2021), and while the tiny neurites of *Drosophila* neurons are shorter than those in the mouse, favoring their three-dimensional reconstruction, their caliber (typically = 0.2 μm) is finer, making comprehensive reconstruction in the z-axis problematic using ssEM.

**FIGURE 2:**
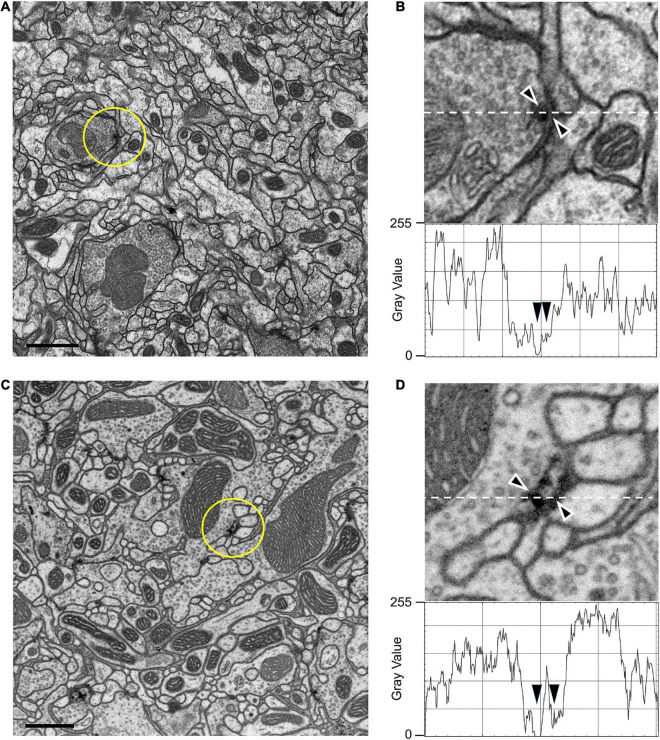
TEM **(A,B)** and FIB-SEM **(C,D)** images compared. **(A)** TEM image from series of 50-nm sections post-stained from the mushroom body calyx reported in Butcher et al. (2012); image resolution is 3.7 nm per pixel. **(B)** Enlargement from **(A)** to show the ultrastructure of a synapse and membranes. Image is scanned in the lower panel to show the gray-scale values for staining, especially for the synapse and synaptic membranes (arrowheads). **(C)** Compare image quality with high-resolution 4-nm per pixel FIB-SEM image of the protocerebral bridge (see also Supplementary Video 2). **(D)** Representative individual synaptic profile and synaptic membranes from **(C)** are indicated by arrowheads in **(D)**, pointed to the T-bar presynaptic ribbon and synaptic membranes. The image is scanned along the interrupted white line to show the gray scale value through organelles. Arrowheads for scan line indicate the electron density of the selected synapse and its membranes. Note that the contrasts of the synaptic T-bar and membrane density in the FIB-SEM image **(D)** both match those from TEM **(B)**. Scale bars **(A,C)** 1 μm.

Overcoming these problems, FIB-SEM (Knott et al., 2008; Xu et al., 2017, 2020) is the preferred method to image *Drosophila* neuropile. Not only does it circumvent the supreme technical skill required to cut extended series of ultrathin sections for serial-section EM (ssEM), but also z-axis resolution is not limited by section thickness. An additional advantage is that z-axis resolution can be adjusted to equal that in x and y (typically 8 nm for FIB-SEM for x, y, and z) compared with TEM (4 nm in x, y and > 40 nm in z: Zheng et al., 2018; Figure 2). FIB-SEM thus provides the means to collect isotropic 8 nm image stacks well suited to reconstruct the slender neurites of *Drosophila* (Hayworth et al., 2015; Takemura et al., 2015; Xu et al., 2017; Shinomiya et al., 2019; Scheffer et al., 2020). Providing an ideal approach to that task, this method has been adopted at the Janelia Campus of HHMI in an intensive effort to derive the entire connectome of a fly’s brain, one that can be comprehensively mined for circuit information (e.g., Takemura et al., 2017; Horne et al., 2018; Scheffer et al., 2020).

EM resolution is required to see synaptic organelles, and the methods for fixing and staining brain tissue in *Drosophila* are well established (e.g., Meinertzhagen and O’Neil, 1991; Yasuyama et al., 2002; Prokop, 2006; Schürmann, 2016); but these have changed little in 50 years, and moreover are not well suited to FIB-SEM imaging. Here, we report various methods that we have developed within the last decade to fix and stain the sub- and supraesophageal regions of the *Drosophila* brain (Figure 1). Together these regulate the segmental ganglia of the ventral nerve cord (VNC), the conduit for much of the brain’s biological output, motor behavior (Niven et al., 2008). Our methods are adapted to automate the segmentation of neurons in both ganglia and VNC, to identify the synaptic profiles between such neurons, and especially to increase FIB-SEM’s formerly slow rate of imaging.

## Materials and methods

### Animals and main steps

As specimens we used Canton-S G1 × w^1118^ wild-type ∼5-day adult *Drosophila melanogaster* maintained at 23–25^°^C on standard fruit fly medium. To prepare *Drosophila* brain tissue specifically to image the entire *Drosophila* brain by FIB-SEM we developed a number of general methods (Table 1), each offering an improvement over the previous one, and we report only our final method in the Results, even though previous methods provide alternative advantages for different aims. We used conventional primary fixation according to the protocol of Takemura et al. (2013) for ssEM and modified this in one of three ways to enable us to minimize the time required for FIB-SEM of an entire *Drosophila* brain. Chief among these, we adopted the hot-knife method (Hayworth et al., 2015) to view several such volumes from successive 20 μm slices imaged in parallel in different machines, and subsequently stitched these to generate a single volume. Each slice comprised 2500 8-nm FIB-SEM images.

**TABLE 1:** *Drosophila* sample preparation procedures and results.

		Methods		
	
Procedure and results	1. HPF-FS	2. PLT-LTS	3. PLT-LTS heavy metal enhancement	4. PLT-LTS progressive heavy metal enhancement

Tissue dissection Pre-fixation	200 μm Vibratome slices	Brain tissue dissect out with metal collar	CNS dissect out with metal collar	Brain dissect out with/out metal collar
Pre-fixation	2.5%GA + 2.5% PFA, RT, 15 min	2.5%GA + 2.5% PFA, RT, 2 h	2.5%GA + 2.5% PFA, RT, 2 h	2.5%GA + 2.5% PFA, RT, 2 h

Post-fixation Heavy metal enhancement	HPF cryo-fixation	0.5% OsO_4_, 30 min, 4°C, W 0.5% UA, 30 min, 0°C, W	0.5% OsO_4_, 40 min, 4^°^C, W no wash, change to 0.8% K ferrocyanide, 2 h, 0°C + 0.5% UA, 30 min, 0°C; W lead aspartate, 4°C overnight; or 4 h RT W 1% OsO_4_, 20 min, 0°C	1% OsO_4_, 40 min, 4°C, W no wash, change to 1.5% K ferrocyanide, 1.5 h, 0°C + 30 min RT; W 1% Tch, 15 min, RT; W, 2% OsO_4_, 30 min, RT; W 0.5% UA, 30 min, RT, W; lead aspartate, 30 min, 55^°^C, and 1 h, RT, W

*en bloc* staining and dehydration	FS: 95% acetone, 1% OsO_4_, 0.2% UA, 1% methanol, 38 h -90°C 14 h -20°C	PLT: acetone, 1% OsO_4_, 0°C to -25°C LTS: 97% acetone with 1% OsO_4_ and 0.2% UA, 30 h, -25°C	PLT: ethanol, 0°C to -25°C LTS: 96% ethanol with 1% OsO_4_ and 0.2% UA, 30 h, -25°C	PLT: ethanol 0°C to -25°C LTS: 96% acetone with 0.2% UA, or 96% ethanol with 1% PTA 30 h, -25°C

Infiltration Embedding	Acetone Durcupan	Propylene oxide Poly/Bed 812	Propylene oxide Poly/Bed 812	Acetone Durcupan

		Summary		

Hot knife cutting	Not suitable	20 μm slices	25 μm slices on BSA en coating tissue	Not suitable

FIB-SEM 8 nm imaging scan rate	1.25 MHz	1.25 MHz	2.5 MHz	10 MHz

FIB-SEM 8 nm imaging speed	20 × 10^3^ μm^3^ per day	20 × 10^3^ μm^3^ per day	40 × 10^3^ μm^3^ per day	150 × 10^3^ μm^3^ per day

FIB-SEM data collection	Medulla, lobula, lobula plate; α-lobe; antennal lobe	Hemi-brain (central complex, mushroom body and more)	Central nervous system (CNS)	Medulla 1st instar larva CNS

LTS, Low temperature staining; W, washing (3 × 10 min). Hot knife cutting properties improved by infiltration in propylene oxide not acetone, and Epon (Poly/Bed 812, Ted pella) instead of Durcupan.

### Method (1) HPS-FS

In the first modification we applied *High Pressure Freezing (HPF) after primary fixation*. The fly’s fixed brain was sliced in a custom-made dissection collar (Supplementary Figure 1A) mounted on the slicing base of a Vibratome. We cut 200 μm slices using a Leica Vt1000 Vibratome (Supplementary Figure 1C); the slices were fixed in 2.5% glutaraldehyde (GA) + 2.5% paraformaldehyde (PFA) for 10–15 min, transferred to 25% aqueous bovine serum albumin (BSA) for a few minutes, and then loaded into a 220 μm deep specimen carrier sandwich, and high-pressure frozen in a Wohlwend HPF Compact 01 High Pressure Freezing Machine (Wöhlwend GmbH, Sennwald, Switzerland). This arrangement of specimen carrier sandwich (Supplementary Figures 2A–C) was chosen instead of a two-hat carrier, widely used in the field for large samples (Murk et al., 2003; McDonald, 2009). After freeze substitution (FS), slices (Figures 2D,E) were embedded in Durcupan (ACM Fluka) epoxy resin (Shinomiya et al., 2019; Xu et al., 2017; Horne et al., 2018; Takemura et al., 2017), in preparation for FIB-SEM (Knott et al., 2008; Xu et al., 2017). The choice of Durcupan is empirical, based on the superiority of this epoxy to Epon in having fewer streaks after imaging (Xu et al., 2017). On the other hand, HPF-FS samples do not cut well for ssEM or during trimming, and to avoid its use we therefore mostly discontinued this freezing method and developed a method for chemical fixation using dehydration by progressive lowering of temperature (Hayworth et al., 2015, see Method 2).

### Method (2) PLT-LTS

**FIGURE 3:**
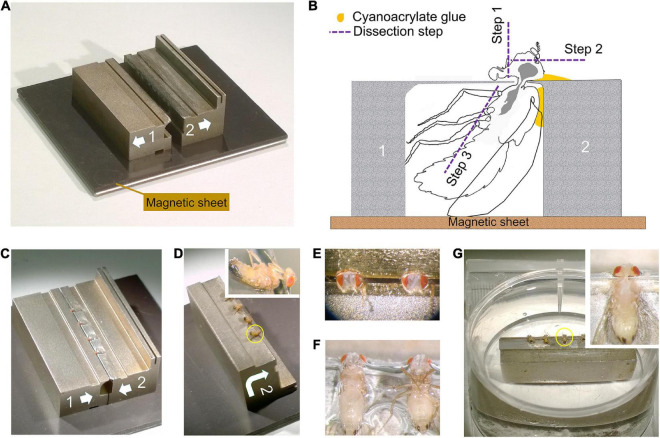
Dissection collar modified for dissecting the entire CNS (see Supplementary Video 1). (A) Two half stocks (1 and 2) arranged to form a pillory on a magnetic base for quick assembly. Quick release shown as arrows on each stock (also in C,D). Stock 2 has an elevated bar for forceps to grip and transfer the stock. (B) Outline of fly in a dissection pillory, held captive at its neck by thin metal shim, to show the position of Loctite glue and dissection steps 1–3. (C) Four flies with protruding heads, loaded and glued into assembled pillory as in (B). (D) Stock 2 separated from its partner stock 1 and turned over, with flies glued by the cuticle of their dorsal thorax as in (B). Inset: Enlarged view of one fly. (E) Two heads in saline after dissection step 1 and 2 in (B) to remove the proboscis and frontal head cuticle together with antennae. (F) Showing the pinioned flies turned over from (E) and transferred to saline, with left-side legs removed as in step 3 in (B). (G) The pinioned flies on stock 2 in saline or PB buffer in tissue culture dish. Inset: Single fly with legs and abdominal cuticle removed to expose the entire CNS (brain and VNC) as shown in the yellow circle in (G).

The fly brain was dissected out by using a metal dissection collar (see Figure 3), then given primary fixation in 2.5% GA + 2.5% PFA in 0.06 M phosphate buffer (PB) for 2 h at 22^°^C, then washed 3 × 10 min in 0.06 M PB followed by cacodylate buffer. Specimens were next exposed for 30 min to 0.5% osmium tetroxide in 0.05 M cacodylate buffer. Then the following procedure was adopted using a protocol we have reported previously in which brains are fixed chemically and processed using dehydration by *progressive lowering of temperature* (PLT) (Supplementary Table 1) also referred to as C-PLT (Hayworth et al., 2015), which reveals synapses having high-contrast organelles. In our current method for adult *Drosophila*, we changed the buffer from 0.1 to 0.06 M, which we have found decreased the electron density of the cytoplasm. We found this decrease by examining multiple specimens, and despite some individual variation between these.

### Method (3) PLT-LTS heavy metal enhancement

In addition to PLT we have employed *heavy metal contrast enhancement*, an improved protocol for *Drosophila* brains that yields an excellent compromise between optimal contrast, sectioning speed, and morphological preservation for FIB-SEM, and is also compatible with hot-knife slicing. This method yields high overall electron contrast for membranes and other cellular structures, but a relatively lower contrast for synapses. After dissection (see Figure 3) and primary fixation as in Method 2, we could either coat the dissected CNS with BSA in order to undertake hot-knife slicing, or without coating, and then wash the specimens for 3 × 10 min in PB and then cacodylate buffer, post-fix them in 0.5% osmium tetroxide in 0.05 M sodium cacodylate buffer, and finally treat them with 0.8% potassium ferricyanide in buffer for 2 h at 4^°^C. After washing in water, we incubated the tissue in 0.5% aqueous uranyl acetate (UA) for 30 min at 4^°^C followed by *en bloc* staining in lead aspartate at 4^°^C overnight, or for 4 h at 22^°^C, and then after further washing in water, for 20 min in 0.8% OsO_4_. For PLT, we placed specimens in a Leica AFS freeze-substitution chamber and dropped the temperature from 4 to -25^°^C, and increased the concentration of acetone or ethanol for 20 min in each of 10, 30, 50, 70, 80, 90, and 97% (Supplementary Table 1). *En bloc staining* and further osmication used a cocktail of 1% osmium tetroxide and 0.2% UA in 97% acetone or ethanol at -25^°^C for approximately 30 h, warming to 22^°^C for final dehydration, then infiltration in acetone or propylene oxide with Epon or Durcupan (Hayworth et al., 2015; Xu et al., 2017). This protocol is current and has been used to analyze the connectome of half a female fly’s brain. For method 3 we did not use osmium-thiocarbohydriazide-osmium (OTO) because this resulted in preparations with inferior cutting properties.

**FIGURE 4:**
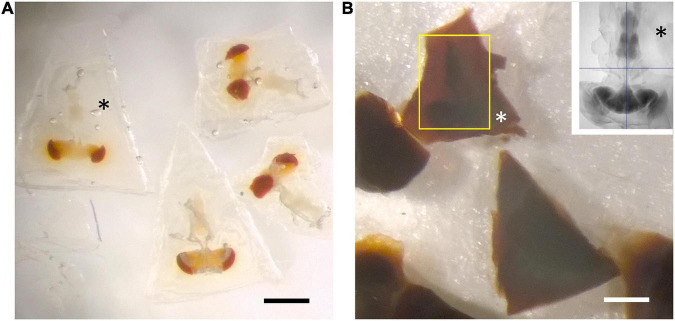
BSA coating of dissected brain. (A) Fixed specimens in trapezoidal blocks after coating with BSA. (B) Osmication and staining with metal salts darkens the BSA, but leaves the brain’s outline still visible (yellow rectangle). Different zones in the surrounding BSA reveal successive additions of the cross-linked protein. Inset: soft X-ray tomogram of a single CNS. Asterisks in all images show the BSA coat in different stages of processing. All scale bars: 500 μm.

To image the entire CNS using FIB, we first needed to cut the preparation into 20–30 μm slices that could be individually handled. For this we improved the hot knife cutting properties using a custom-made ultrathick sectioning microtome (Hayworth et al., 2015). To improve the cutting properties of the brain and preserve the integrity of its external surface, which is easily distorted, we developed a new method, enclosing the brain in a 25% solution of BSA in 0.06 M phosphate buffer (PB) after primary fixation. This process relies on cross-linking the BSA after aldehyde fixation. We do this by placing three drops in a Petri dish, the first containing 25% BSA, the second containing fixative, and the third buffer wash (PB). The brain is first transferred from the BSA (drop 1) to the fixative drop (drop 2) to coat it with a thin layer of fixed protein, and next transferred to the buffer wash drop (drop 3), then back to the BSA drop. This sequence was sometimes repeated, to ensure that a thin layer of BSA adhered to the ventral surface of the specimen. Using this sequence, we added a drop of fixative on top of the BSA droplet containing the sample, and waited until the BSA polymerized. We then cut the polymerized BSA into a regular trapezoid containing the orientated sample at its center (Figure 4A). The inclusion is then carefully lifted and transferred to a droplet of buffer wash. After osmication, heavy metal staining and further processing, the BSA coating layer darkened (Figure 4B). We used soft X-ray tomography to provide a means to view the sample’s profile and orientation from its opaque BSA coating (Figure 1).

### Method (4) PLT-LTS progressive heavy metal enhancement

For contrast, we used OTO to provide high contrast, because we were not concerned about the quality of cutting. We used this method to image the larval brain, which is too small to require hot-knife subdivision. After tissue dissection and pre-fixation as in methods 2 and 3, we osmicated tissue in 1% OsO_4_. This was followed without washing by 1.5% K ferrocyanide, then a complete wash and finally, a transfer for 15 min to 1% thiocarbohydrazide at 22^°^C, followed in turn by a complete wash then 2% osmium for 30 min at 22^°^C. After osmication we stained in lead aspartate for 30 min at 55^°^C, followed by 1 h at 22^°^C. The tissue was then dehydrated using the PLT method as in Method 3, followed by low temperature *en bloc* staining in either 0.2% uranyl acetate in acetone, or 1% EPTA in 97% ethanol. Specimens were infiltrated and embedded as for methods 1–3 in Epon or, in the case of FIB, Durcupan.

## Results

We present a consolidated method for FIB-SEM of the *Drosophila* brain, based on a number of protocols (Table 1), each compiled from multiple parametric repetitions. Together with earlier methods (Figure 2A), these have taken a decade to develop and perfect. Central to them were the development of new microdissection protocols (Figure 3 and Supplementary Figure 1), improvements in the heavy metal staining of the brain that support faster rates of FIB-SEM imaging, and the exact targeting of specific regions using X-ray tomography of embedded stained brains.

### General features of FIB-SEM images

The general features of FIB-SEM images obtained using the updated fixation and staining method we present below are first authenticated against conventional images obtained with TEM. To make this comparison valid, we first needed to capture FIB-SEM images at higher resolution (4 nm/pixel in x,y,z, Figures 2C,D) than we routinely used (8 nm/pixel) to be more nearly comparable to the TEM image of the same brain region, for which we illustrate a region of the protocerebral bridge (Figures 2C,D). Cell and organelle profiles visible from TEM are all immediately recognizable in the FIB-SEM image, and indistinguishable using either imaging method at the magnifications chosen. Importantly for segmentation, cell membranes and synaptic profiles are all clearly visible. Pre- and postsynaptic elements were both more electron-dense than with conventional TEM methods and post-staining with uranyl acetate and lead citrate (compare Figures 2B,D). The FIB-SEM illustrated, 4 nm/pixel in x,y,z (Figure 2C), has a higher resolution than that (8 nm) at which neurons were routinely segmented, however, each reconstructed voxel thus having a volume 2^3^ = 8 times larger. Synaptic sites (Figures 2B,D) could be clearly detected semi-automatically from their increased electron density (Huang et al., 2018), with typically a single T-shaped presynaptic density (or T-bar), opposite which sit a number of postsynaptic processes. In addition to synapses, mitochondria are well preserved and suitable for automated classification and segmentation (Scheffer et al., 2020) at 8 nm/pixel, and 3D reconstruction to reveal mitochondrial internal structure at 4 nm/pixel (Supplementary Figure 4).

### Microdissecting the fly’s brain

To prepare adult *Drosophila* brains for imaging, various previously reported dissection methods (Meinertzhagen, 1996; Wolff, 2011) are mostly too rudimentary. To visualize neurons that arborize not only in the dorsal supraesophageal brain but also in the subesophageal ganglion and VNC, we imaged each part in parallel to reconstruct both arbors of single neurons. For this, we developed a method to microdissect and fix the two ganglia of a single brain intact, together with their corresponding cervical connectives. This required that we dissect the *Drosophila* brain by holding the head in a custom machined metal collar (Heisenberg and Böhl, 1979) and then transfer the ensemble to primary fixative. The yield of well-preserved brains is not high and successfully increased only by means of such collars. About four heads each held in a single collar are together transferred to primary fixative within about 5 min. In most reports in the literature, the lamina is simply torn off, so that the brain’s outer margin is the medulla cortex, but in our improved methods we use careful dissection to retain the lamina, which offers its own merits as a model neuropile (Meinertzhagen and O’Neil, 1991; Meinertzhagen and Sorra, 2001). In its current application, the method is further modified into a two-stage dissection that enables us to preserve intact the supraesophageal brain together with the subesophageal, and thus enables us to reconstruct in their entirety those neurons that arborize in the neuropiles of both regions. Flies are held in a modified collar consisting of two halves held together on a magnetic base (Figure 3). This assembly is used to dissect the dorsal and ventral brains attached, in two steps. First, the dorsal cuticle of the head and thorax is attached to one side of the collar with a tiny amount of cyanoacrylate glue (Loctite 404) (Figures 3B–D). The exact amount is important and needs to be determined empirically. The proboscis and frons cuticle of the head are removed in a drop of saline (Olsen et al., 2007). Then the unglued side of the collar is removed and the attached side turned into the horizontal plane and transferred into a Petri dish in a pool of saline (Wilson and Laurent, 2005), and the legs removed (Supplementary Video 1). Next, the assemblage comprising the half collar attached to the partially dissected fly is transferred to primary fixative. Further steps are undertaken after 2 h of fixation in 2.5% glutaraldehyde and 2.5% paraformaldehyde in 0.06 M phosphate buffer. The second stage of dissection is undertaken in the same buffer. The head cuticle is removed, the collar turned 90^°^ and the subesophageal ganglion and VNC dissected out. Even though the specimen is now fixed, the cervical connectives are structurally very weak after fixation and the specimen must be handled with great care to avoid fracturing its axons, especially those of neurons that arborize in both ganglia. Despite these precautions, occasional dark profiles reflect the inevitable collateral damage of degenerating axons especially amongst the distal ends of afferent axons severed during the process of dissection.

### Speeding FIB-SEM: Parallel imaging of hot-knife slices

**FIGURE 5:**
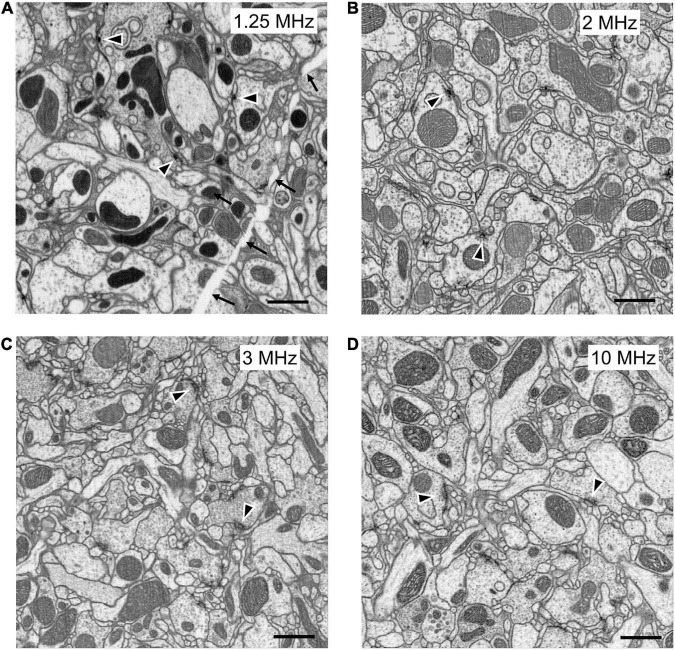
FIB-SEM images, from samples prepared with different methods at different scan rates. All produce images of comparable quality, collected at different imaging speeds. (A) High-pressure freezing with freeze substitution, HPF-FS to compare with (B) progressive lowering of temperature and low-temperature staining (PLT and LTS). (C) Heavy metal enhanced PLT method scanned at 1.5 times the rate (3 MHz) as in (A,B). (D) PLT-LTS progressive heavy metal enhancement, imaged more rapidly than the preceding (C), according to Method 4 in Table 1. Synaptic profiles (arrowheads) are clear in all panels but unavoidable cracks (arrows in A) appear during HPF-FS specimen preparation. Scale bars: 1 μm.

**FIGURE 6:**
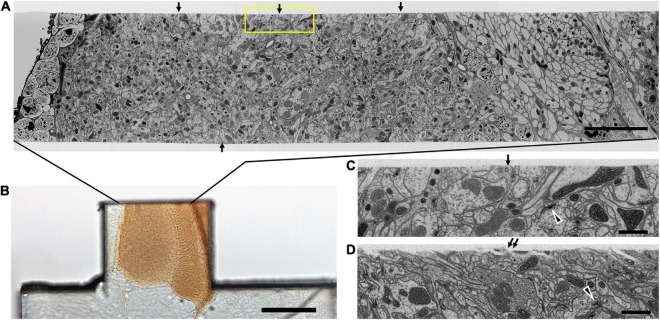
Quality of the cut surface of hot-knife slice. (A) Entire FIB-SEM image of the medulla neuropile of a 20 μm thick hot-knife slice shown in (B). (B) Light micrograph of 20 μm slice of optic lobe. (C) Enlarged area of one side of the cut surface, shown enclosed in yellow box in (A). Arrows in (A) show the smoothness of the cut surface of the slice. Arrowhead in (C,D) shows selected synaptic profile. Arrows in (C,D) show the surface of the hot-knife slice, smooth (C) but occasionally rough (D). Scale bars: 10 μm (A); 100 μm (B); 1 μm (C,D).

Until now, FIB-SEM has been the slowest, and most costly imaging step in fly connectomics and could capture only small specimen volumes. For example, using FIB-SEM at an 8 nm resolution the scan rate is only 0.3 MHz, covering a daily volume of just 6 × 10^3^ μm^3^ per day (Table 1, Method 1; Figure 5A). Even though the dimensions over which a block can be milled, 400 × 300 μm (x, y) and = 400 μm in z, could potentially include those of the fly’s entire brain, the area over which we could routinely collect a high-dose image stack using FIB-SEM without severe milling artifacts is far smaller than this (Xu et al., 2017). Meeting the need for increased ease and speed, during the last decade at Janelia we have developed successive generations of methods. In a first step, borrowed from an earlier precedent with light microscopy (McGee-Russell et al., 1990), we used the so-called hot-knife protocol to cut ultra-thick (∼20 μm) slices (Hayworth et al., 2015; Figure 6B) of an Epon embedded brain coated with Durcupan. The choice of Durcupan was empirical, based on the superiority of this epoxy over Epon in having fewer streaks after FIB-SEM imaging (Xu et al., 2017). With the hot-knife method we could distribute the task of concurrent imaging amongst several slices, each imaged in a different machine. The female half-brain we report comprised about 13 20μm slices in a sagittal plane with a total imaged volume of up to ∼1.6 × 10^7^ μm^3^, and we stitched consecutive image stacks to yield a final volume (Hayworth et al., 2015). For the entire CNS the volume comprised parts of 27 25μm sagittal slices through the dorsal brain and 26 cross sections through the ventral nerve cord, much larger than the female half brain. Even using the hot-knife slicing strategem and imaging voxels at 8 nm, FIB-SEM typically covers not more than about 30 × 10^3^ μm^3^ per day per FIB-SEM machine (Table 1, Method 2; Figure 5B), and thus initially was painfully slow. In parallel with hot-knife slicing and to increase FIB-SEM imaging speed yet further, we developed revised staining methods to a level that would enhance overall image contrast, and thereby support increased FIB-SEM imaging speeds. Our new staining method had to provide enhanced contrast optimally suited not only to detecting synapses but also simultaneously enhancing tissue and membrane contrast. For this, we developed a heavy metal method to enhance staining, which has sped up the imaging speed to 50 × 10^3^ μm^3^ per day per machine (Table 1, Method 3; Figure 5C).

The increased contrast of these combined methods enabled us automatically to segment the profiles of neurons far faster than initially, while imaging at rates that now match those of ssEM (Xu et al., 2017). In consequence, imaging times are similarly reduced, thus saving in parallel the cost of expensive FIB-SEM imaging time. Moreover, although we stitched a few (up to 5) tiles per imaging plane, the isotropic image stacks so compiled did not require labor-intensive construction as montages, only that the image collected from each hot slice be stitched to that of its neighbors in the stack.

### New *en bloc* staining methods

In our first attempts to image an entire fly’s brain using FIB-SEM we encountered successive problems, for which we developed a number of new methods (Table 1). To obtain good brain preservation, especially with clear synaptic densities, we first used high-pressure freezing (HPF, Supplementary Figure 2 and Table 1, Method 1) as a comprehensive method to analyze the entire *Drosophila* brain from successive slices. HPF and freeze substitution (FS) is considered the best approach for achieving close-to-native-state brain ultrastructure (Korogod et al., 2015; Tsang et al., 2018), but this technique works best with samples ≤ 200 μm in thickness. Although HPF-FS of *Drosophila* brain subvolumes was achievable and provided specimens with good morphology and image contrast (Takemura et al., 2015, 2017; Horne et al., 2018; Shinomiya et al., 2019), larger brain blocks prepared with HPF-FS developed cracks and lacked the cutting properties for the hot-knife protocol that we required to sample large volumes of the fly’s brain. To achieve close-to-native-state ultrastructure in whole brains, we therefore turned to the progressive lowering of temperature (PLT) technique. PLT is a dehydration scheme whereby organic solvent concentration is increased at progressively lower temperatures (0 to -50^°^C); it was first developed to preserve membrane structure and protein antigenicity in combination with low temperature embedding resins (Roth et al., 1981; Armbruster et al., 1982; Carlemalm and Kellenberger, 1982; Carlemalm et al., 1985; Möbius, 2009). By combining PLT dehydration with *en bloc* low temperature staining (LTS) and further osmication (Table 1, Method 2), we preserved all the favorable features of high pressure freezing, and cured the problem of cracked blocks, staining T-bars at synaptic sites dark, as well as enabling us to slice the brain using the hot-knife method (Hayworth et al., 2015). Nevertheless the overall image speed was still slow. For example, using the PLT-LTS protocol above, FIB imaging took ∼80 days per slice, but without incurring specimen cracks, while retaining good hot-knife cutting properties to allow simultaneous parallel imaging of multiple slices. To improve imaging speed we also increased tissue contrast by means of supplementary heavy metal enhancement, using potassium ferricyanide and lead aspartate (Table 1, Method 3).

Despite these advantages, the heavy metal exacerbates the poor cutting property of the brains for thick sectioning. We overcame this problem by first coating the brain with bovine serum albumin (BSA, Figure 4A). This provides a smooth surface to the cut edge (Figures 6A,C,D) that was useful later in improving the registration of cut surfaces between consecutive slices, as well revealing structural features that provide SEM focus to be optimized before the tissue itself is milled. This method proved superior to that of Hildebrand et al. (2017) in diminishing the gap between BSA and the fly brain tissue, and in providing a more extensive surface of contact. However, a countering disadvantage was that the BSA darkens and obscures the outline of the enclosed fly brain (Figure 4B). To overcome this problem, therefore, we imaged the tissue with soft X-ray tomography (microCT) prior to FIB-SEM, a combination of methods that enabled us to select the desired brain region with great accuracy.

To obtain this improved staining, fly heads were dissected and prepared in a metal collar, as given above (Figure 3), pre-fixed in a mixture of 2.5% of each of glutaraldehyde and paraformaldehyde at room temperature (22^°^C) for 2 h, and then post-fixed in 0.5% OsO_4_ for 40 min at 4^°^C, followed by three 10-min washes in water; then heavy metal enhancement in 0.8% K ferrocyanide for 2 h at 0^°^C and 0.5% uranyl acetate, 30 min at 0^°^C; a wash, and then staining in lead aspartate overnight at 4^°^C; followed by a distilled water wash, and then 20 min in 0.8% OsO4 at 0^°^C. This is lastly followed by PLT-LTS as in Method 2 (Table 1) and embedment in Epon or Durcupan.

In addition to applying this protocol to adult flies, we also developed a method by combining ferrocyanide reduced osmium-thiocarbohydrazide-osmium (R-OTO: Willingham and Rutherford, 1984) to PLT-LTS that enabled us to image the brains of first-instar larval *Drosophila*. The larval brain is smaller, and thus unlike the adult brain, did not require hot-knife slicing. This progressive heavy metal enhancement method (Table 1, Method 4, Figure 5D) applied to both larva and adult used the advantages of Method 3 while increasing tissue contrast; in combination with the brain’s small size it enabled us to collect an image stack with a high FIB-SEM imaging scan rate of 10 MHz, to achieve 200 × 10^3^ μm^3^ per day per machine at 8 nm resolution (Table 1, Method 4), > 30 times faster than Method 1.

### X-ray tomography

To locate regions of interest, we routinely employ soft X-ray tomography of osmicated specimens viewed *en bloc*, using an Xradia Versa 3D XRM-510 to preview the specimen and select out those specimens having cracks, vacuoles or distortions that would have wasted valuable imaging time on flawed specimens. This important step also enables us to identify the coordinates of imaged structures prior to trimming the block to a specific depth for FIB milling (Takemura et al., 2017), in a combination of methods that enabled us to select the desired brain region with great accuracy. Both selections, of the region of interest and its depth, offer considerable prospective savings against wasting time to mill and image through unwanted sample areas, during the lengthiest but most valuable, and costly step in the entire process. Executing this step requires some experience, however, Scrupulous preservation and integrity of the brain is required because of the large time investment in fixation and FIB-SEM imaging made after the initial dissection, and because superior fixation can only be selected at the end of these steps, after a lengthy period of imaging that would otherwise be wasted on an inferior sample.

## Conclusions

Dissection and fixation are the first essential steps to view cells and organelles. In previous studies, dissection of the *Drosophila* brain has generally been minimal, involving only removal of the eye and lamina of each side, and fixation is aided by the brain’s tiny dimensions, < 150 μm along the head’s anteroposterior axis (Peng et al., 2011), and are hence well suited to EM. Most conventional primary fixation methods employ aldehydes, especially primary fixation by the formaldehyde/glutaraldehyde (PFA/GA) mixture with high osmolarity introduced by Karnovsky (1965) > 50 years ago. The advantage of this and other double-aldehyde fixatives is that they provide a universal method that needs no refinement for particular nervous systems, even if many simple invertebrate nervous systems do not in fact fix well with it. *Drosophila* is generally well preserved with aldehyde fixation methods (Meinertzhagen, 1996; Wolff, 2011), but for neuropiles a general problem is to capture neurites as profiles that are round in cross section and well separated from those of their neighbors, well suited to automated segmentation. Most EM using previous techniques preserves many neurites only as flattened and polymorphic profiles, however, a usual condition in published EM images, and makes the continuity of these hard to follow in an image stack. To enhance membrane density, high-pressure freezing and freeze substitution (HPF-FS: Walther and Ziegler, 2002), and ferrocyanide reduced osmium-thiocarbohydrazide-osmium ligand binding (R-OTO: Willingham and Rutherford, 1984), have all been used, but each has its own shortcomings particularly for intact insect brain tissue.

Addressing these deficiencies, we report a number of methods adapted to the analysis of synaptic circuits in the *Drosophila* brain (Supplementary Information). The detailed protocol we present for *Drosophila* incorporates various component methods which, in differing combinations, are likely to suit the fixation of brains in other model species. For example, preliminary TEM screening of mouse brain tissue processed with PLT-LTS reveals well preserved synapses and neuronal processes (Supplementary Figure 5). Individual steps in our protocols, for example BSA coating for hot-knife slicing of entire brains and our dissection protocols, are equally applicable to the connectomics of other species. They are the product of a decade of our development from earlier protocols. Each offers particular advantages, but most important for our purposes, we report a method to improve the imaging speed of FIB-SEM by adopting novel ways to increase specimen contrast, and we apply these to an entire microdissected hot-knife sliced fly’s brain comprising connected sub- and supraesophageal ganglia. Our methods are adapted to a FIB-SEM imaging mode and reliably recover fixed neurons as round cross-sections, suitable for machine segmentation (Parag et al., 2015), with dark synaptic profiles suitable for automated synapse detection (Huang et al., 2018). The numbers of the latter match closely the numbers of those identified by human proof-readers (Shinomiya et al., 2019) and so are considered accurate.

In aggregate our collective methods, those reported here and others developed at Janelia (Supplementary Information Methods), provide a means for semi-automatic segmentation of *Drosophila* neurons and automated synapse detection. In particular, our staining methods now provide an excellent compromise between specimen contrast and accelerated FIB-SEM sectioning speed. Imaging speed may be further enhanced using higher specimen contrast to yield usable images yet more quickly, however; and in the future also possibly by using gas cluster milling (Hayworth et al., 2020) combined with SEM with multi-beam imaging (Eberle and Zeidler, 2018). Even so, many sensory inputs to both brain regions are necessarily removed when their axons are severed, and these leave behind degenerating afferent axons, which yield electron-dense profiles (Supplementary Figure 6). Darkened degenerating axons visible in EM are known to appear with a very rapid onset (Brandstätter et al., 1991) and in our case are thought to signify those axons that were severed, or also possibly simply stretched, during the relatively short period of dissection and immediate fixation.

The rationale for our PLT-LTS method is based on previously reported size measurements in cells prepared for EM. PLT-LTS gives the tissue intense staining and fewer structural alterations than routine dehydration and *en bloc* staining. Using a lower concentration of ethanol (< 70%) during dehydration causes the tissue to swell, whereas with dehydration in absolute ethanol the tissue shrinks (Konwi‘nski et al., 1974). Dehydration at low temperatures can minimize these size and shape changes. We also found that after staining tissue at 0–25^°^C in acetone- or ethanol-based uranyl acetate after routine fixation, the FIB-SEM images showed improved contrast compared with routine staining with aqueous UA at 4^°^C (Supplementary Figure 3). Using acetone gave the best results in tissue contrast but the hot-knife cutting properties were worsened, making a compromise necessary. The PLT-LTS method helps to provide uniform osmication and staining, with less chance of distorting the fine structure. The method works well on the entire adult *Drosophila* brain as well as that of the first-instar larva. To extend the PLT technique this protocol could be further improved by introducing lead acetate, tannic acid, imidazole, phosphotungstic acid, and organic solvent soluble stains into the protocol.

Finally, our method incorporates an important advance in reliably being able to preserve both parts of the CNS intact while these still remain connected, and thus make it possible to image the delicate pathways between the supraesophageal and subesophageal ganglia of the brain and the cells that arborize in both. Preserving the continuity of pathways through the connectives ensures retention of the integrity of descending inputs to the many lineages of subesophageal neurons (Shepherd et al., 2019), as well as complementary ascending pathways. Only by retaining both halves of the brain can cells with neurite arbors in both be preserved complete. An unavoidable consequence of removing the brain from the fly’s head is, even so, that many sensory inputs to both brain regions are necessarily removed when their axons are cut, and these leave behind degenerating afferent axons, yielding electron-dense profiles. These we regard as the small inevitable price to pay for the opportunity our methods provide to identify the brain circuits formed by the majority of intact well-preserved axons that span both brain regions.

## Data availability statement

The original contributions presented in this study are included in the article/Supplementary material, further inquiries can be directed to the corresponding author/s.

## Author contributions

ZL undertook all fixations and EM analyses, prepared the all figures, and helped prepare the manuscript. CX, SP, and HH undertook all FIB imaging. SMP and LS undertook EM analyses and image alignments. PR evaluated ultrastructural preservation and image quality and assisted with EM resources. KS evaluated image quality and reconstructed mitochondria. KH evaluated compatibility with hot knife technique and provide valuable discussions on all methods. GR, IM, and PR prepared the manuscript. All authors contributed to the article and approved the submitted version.

## Supplementary material

Supplementary Figure 1 Dissection collar for *Drosophila* Vibratome slice. (A) Custom-made dissection collar mounted on a Vibratome sample loading base. A slot holds a row of flies captive at their necks, held on a thin metal shim between four reservoirs for saline. (B) Side view of a fly in the collar with its head protruding through the slot, mounted with cyanoacrylate cement (Loctite) and covered in 5% agarose to stabilize the entire head during slicing, when cut at an angle by a thin high-carbon steel Feather double-edge razor blade. The first cut removes the anterior head cuticle, after which a single drop of fixative starts fixation, immediately followed by a second cut, which removes a 200 μm slice. (C) Vibratome slice of five fly heads (circle) in a collar. (D) Enlarged frontal view of three of the heads in the collar (from C).

Supplementary Figure 2 High-pressure carrier for freezing *Drosophila* brains. (A) Custom-made aluminum sandwich carrier for high-pressure freezing fly heads, comprising a machined annulus (2) sandwiched between two hat-shaped plates (1,3). (B) Cross section of sandwich in a, with 200-250 μm Vibratome slice of a fly head (black profile) in specimen annulus (2) supported between the two hat-shaped plates (1,3) coated on their inner faces with lecithin. Final assembled thickness is 1000 μm to fit in the specimen holder of a Wohlwend HPF Compact 01 High-Pressure Freezing Machine (Wohlwend GmbH, Sennwald, Germany) as shown in (C). (D) Samples in specimen annulus (2 in A–C) after polymerization. The hinged top and bottom layers (1,3 in A–C) are removed before freeze substitution. During freeze substitution the medium can substitute from both free surfaces. Specimens are surrounded by filler (20% BSA filler in water), yellow after polymerization. A specimen annulus having a round well provides a larger area for freeze substitution than one with an elliptical well. Specimens are easily removed from the annulus by cutting the latter along one diameter with a single-edge razor blade.

Supplementary Figure 3 Comparison of the contrast in FIB-SEM images using UA staining. Comparison between *en bloc* staining of aqueous UA (A,B) and organic-solvent based UA (C,D) on adult *Drosophila* brains. There are three parts on each figure. Top part shows normalized half of raw image; middle part shows raw data without changing the range of pixel intensity values; bottom part shows “Plot Profile” to display a 2D graph of the intensities of pixels along a blue line within each image. (A,B) Show the tissue *en bloc* staining with aqueous 0.3% UA and 1% UA overnight at 4^°^C with conventional fixation and dehydration procedure. (C,D) Show the tissue staining with 0.3% UA in ethanol and 0.3% UA in acetone in PLT-LTS procedure (see Table 1, Method 2). The overall contrast produced by aqueous UA staining is lower than the staining contrast with UA in ethanol or acetone. Scale bars 1 μm.

Supplementary Figure 4 Mitochondria are well preserved in the *Drosophila* brain after fixation and staining using PLT-LTS. (A,B) FIB-SEM image of mushroom body at 8nm/pixel, (A) mitochondria are well stained, intact (labeled as M), and suitable for automated classification and segmentation as show in (B). (C–E) FIB-SEM image of protocerebral bridge at 4 nm/pixel, (C) mitochondrial internal structure is well preserved and varies between mitochondria in this region of the brain, (D) 3D reconstruction of mitochondrion labeled (*) in (C). (E) 3D reconstruction of more darkly stained mitochondrion showing densely packed cristae. Scale bars 1 μm.

Supplementary Figure 5 TEM image of mouse cortex after fixation and staining with PLT-LTS with heavy metal enhancement. Synapses (arrow), synaptic vesicles (SV) and mitochondria (M) are well preserved. Scale bar 1 μm.

Supplementary Figure 6 TEM image of *Drosophila* optic lobe after fixation and staining with PLT-LTS. Most neuronal processes have light (L) cytoplasm, but some have dark (D). Synapses (arrow) are detectable in light and dark processes in this sample. Scale bar 1 μm.

Supplementary Video 1 https://figshare.com/s/1f66b303e601da6edc78. Steps in the dissection of *Drosophila* CNS (see Figure 3).

1. Load flies into dissection collar (Figure 3C) and glue head and body on the collar (Figures 3C,D)
2. Dissection step 1, 2 and 3 (Figures 3B,E,F)
3. Final step to dissect CNS out (Figure 3G)

Dissection tools:

• Metal dissection collar (customer made)
• Fine forceps
• Feather Razor blades
• Blade Holders & Breakers—Concave-Convex Jaws
• Cyanoacrylate glue.

Supplementary Video 2 High-resolution FIB-SEM stack of sample produced with PLT-LTS. Video of high-resolution 4-nm per pixel FIB-SEM image stack of the protocerebral bridge showing the quality of PLT-LTS sample preservation. Single image in Figure 2C is taken from this stack.
