# Supplementary figures and images for: En bloc preparation of Drosophila brains enables high-throughput FIB-SEM connectomics
Source: Front Neural Circuits. 2022 Dec 16;16:917251. doi: 10.3389/fncir.2022.917251 (PMC9801301; doi:10.3389/fncir.2022.917251)

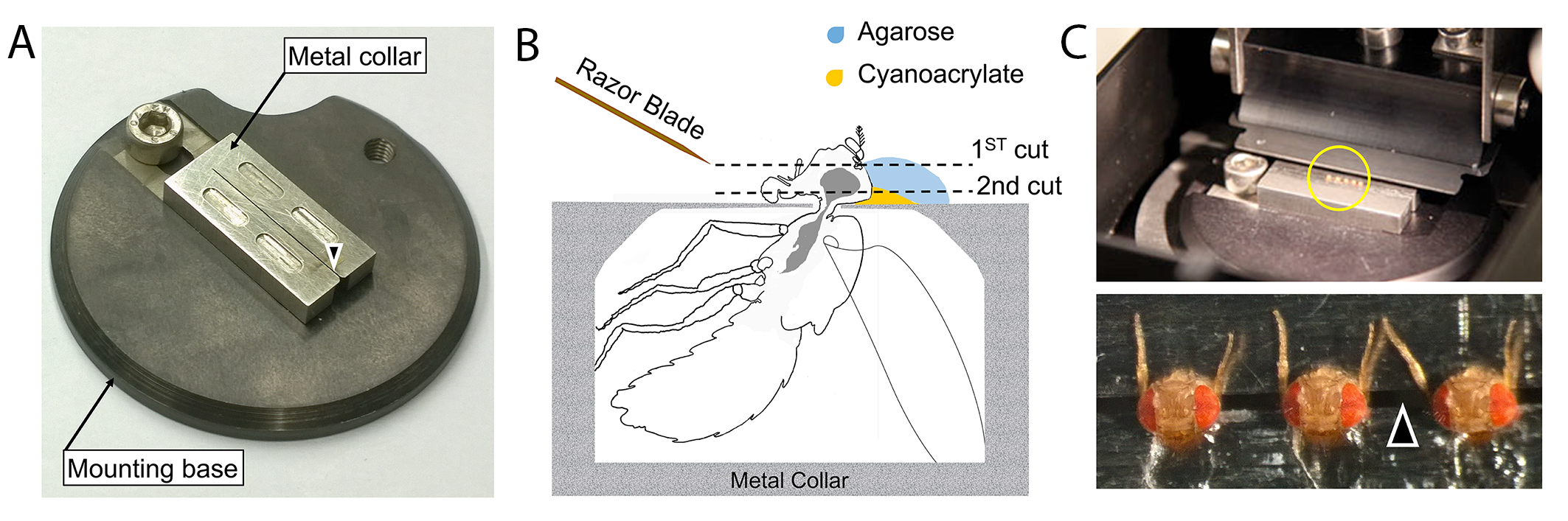

Supplement: Supplementary Figure 1 — Dissection collar for Drosophila Vibratome slice. (A) Custom-made dissection collar mounted on a Vibratome sample loading base. A slot holds a row of flies captive at their necks, held on a thin metal shim between four reservoirs for saline. (B) Side view of a fly in the collar with its head protruding through the slot, mounted with cyanoacrylate cement (Loctite) and covered in 5% agarose to stabilize the entire head during slicing, when cut at an angle by a thin high-carbon steel Feather double-edge razor blade. The first cut removes the anterior head cuticle, after which a single drop of fixative starts fixation, immediately followed by a second cut, which removes a 200 μm slice. (C) Vibratome slice of five fly heads (circle) in a collar. (D) Enlarged frontal view of three of the heads in the collar (from C). [file Image_1.TIF]

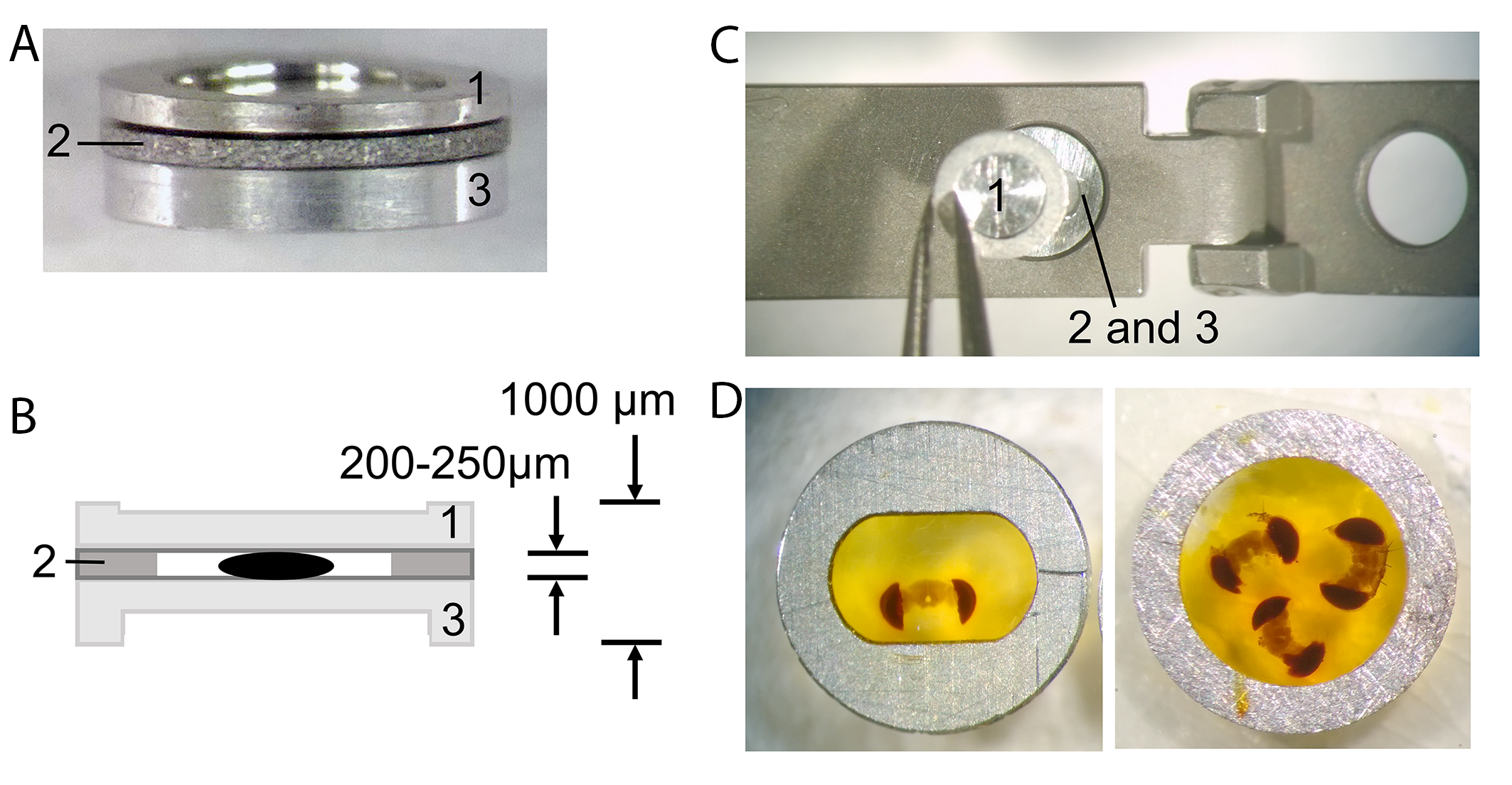

Supplement: Supplementary Figure 2 — High-pressure carrier for freezing Drosophila brains. (A) Custom-made aluminum sandwich carrier for high-pressure freezing fly heads, comprising a machined annulus (2) sandwiched between two hat-shaped plates (1,3). (B) Cross section of sandwich in a, with 200-250 μm Vibratome slice of a fly head (black profile) in specimen annulus (2) supported between the two hat-shaped plates (1,3) coated on their inner faces with lecithin. Final assembled thickness is 1000 μm to fit in the specimen holder of a Wohlwend HPF Compact 01 High-Pressure Freezing Machine (Wohlwend GmbH, Sennwald, Germany) as shown in (C). (D) Samples in specimen annulus (2 in A–C) after polymerization. The hinged top and bottom layers (1,3 in A–C) are removed before freeze substitution. During freeze substitution the medium can substitute from both free surfaces. Specimens are surrounded by filler (20% BSA filler in water), yellow after polymerization. A specimen annulus having a round well provides a larger area for freeze substitution than one with an elliptical well. Specimens are easily removed from the annulus by cutting the latter along one diameter with a single-edge razor blade. [file Image_2.TIF]

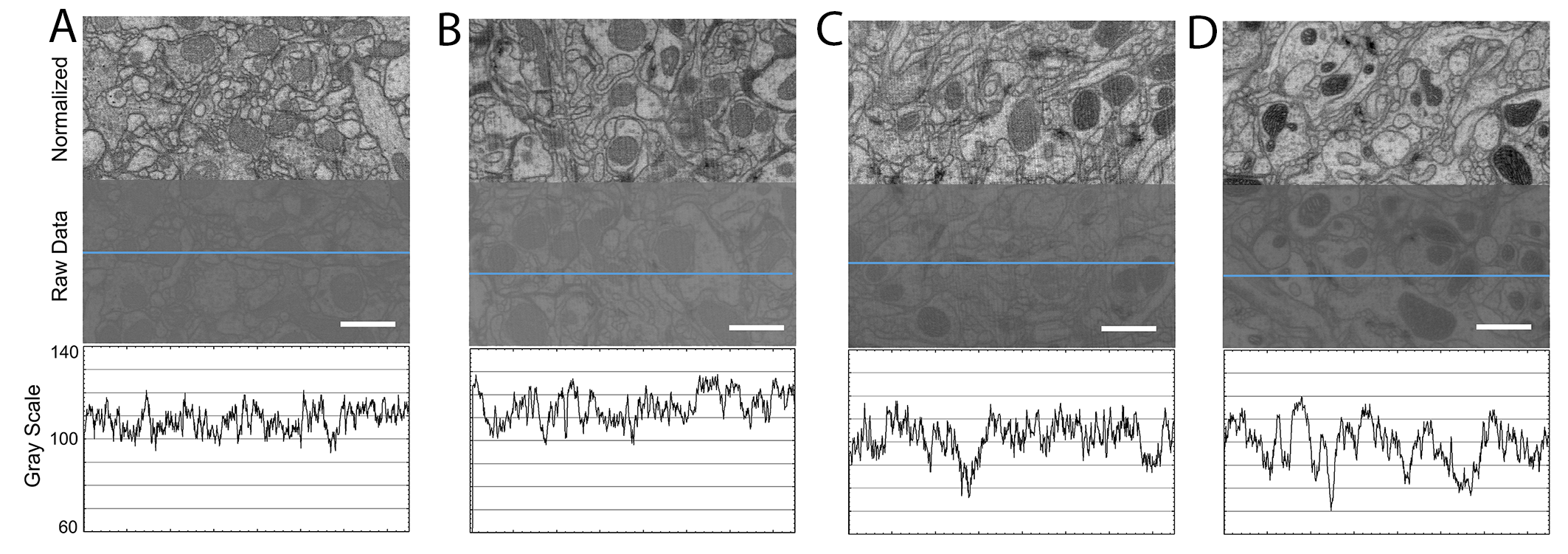

Supplement: Supplementary Figure 3 — Comparison of the contrast in FIB-SEM images using UA staining. Comparison between en bloc staining of aqueous UA (A,B) and organic-solvent based UA (C,D) on adult Drosophila brains. There are three parts on each figure. Top part shows normalized half of raw image; middle part shows raw data without changing the range of pixel intensity values; bottom part shows “Plot Profile” to display a 2D graph of the intensities of pixels along a blue line within each image. (A,B) Show the tissue en bloc staining with aqueous 0.3% UA and 1% UA overnight at 4°C with conventional fixation and dehydration procedure. (C,D) Show the tissue staining with 0.3% UA in ethanol and 0.3% UA in acetone in PLT-LTS procedure (see Table 1, Method 2). The overall contrast produced by aqueous UA staining is lower than the staining contrast with UA in ethanol or acetone. Scale bars 1 μm. [file Image_3.TIF]

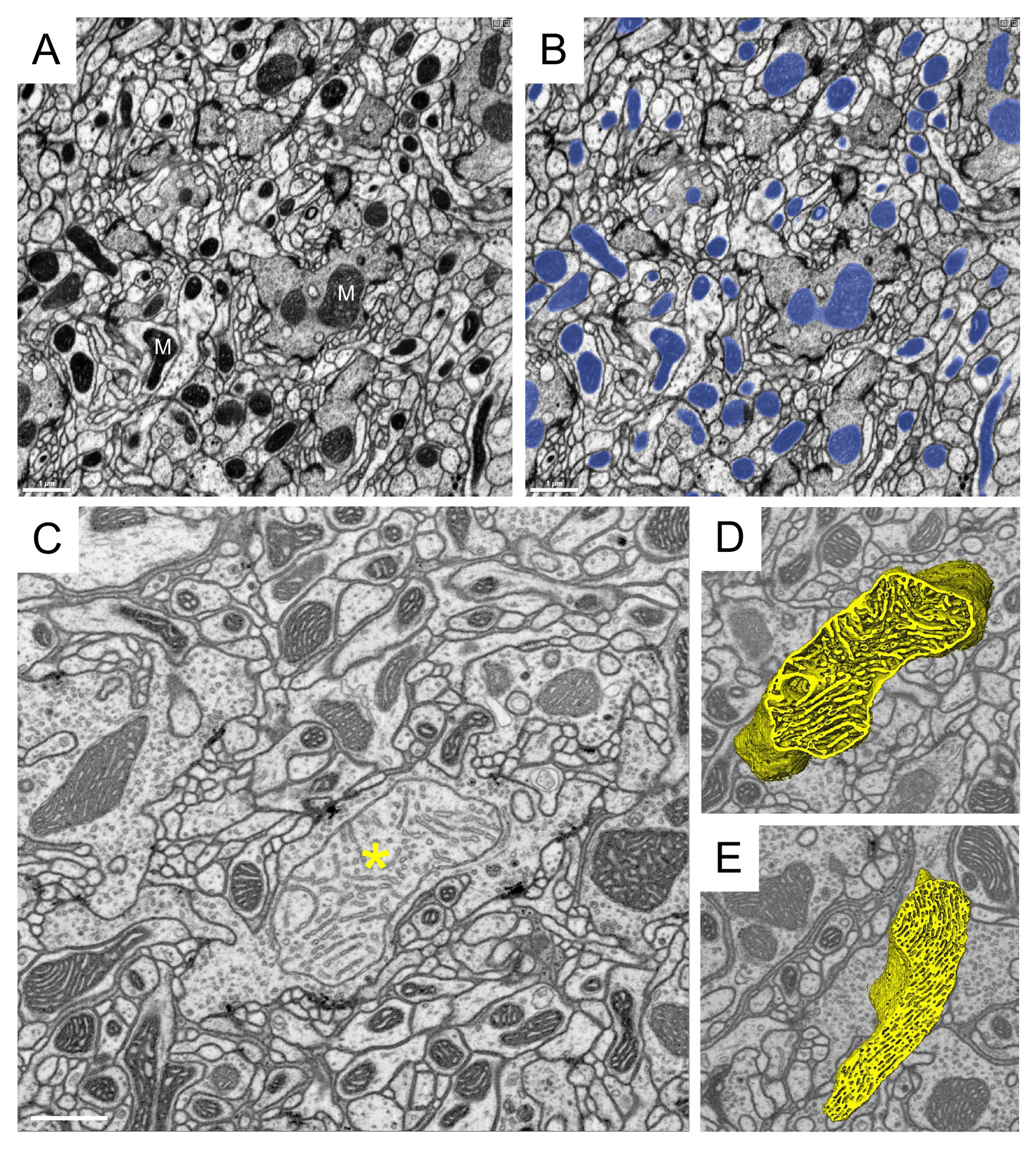

Supplement: Supplementary Figure 4 — Mitochondria are well preserved in the Drosophila brain after fixation and staining using PLT-LTS. (A,B) FIB-SEM image of mushroom body at 8nm/pixel, (A) mitochondria are well stained, intact (labeled as M), and suitable for automated classification and segmentation as show in (B). (C–E) FIB-SEM image of protocerebral bridge at 4 nm/pixel, (C) mitochondrial internal structure is well preserved and varies between mitochondria in this region of the brain, (D) 3D reconstruction of mitochondrion labeled (*) in (C). (E) 3D reconstruction of more darkly stained mitochondrion showing densely packed cristae. Scale bars 1 μm. [file Image_4.TIF]

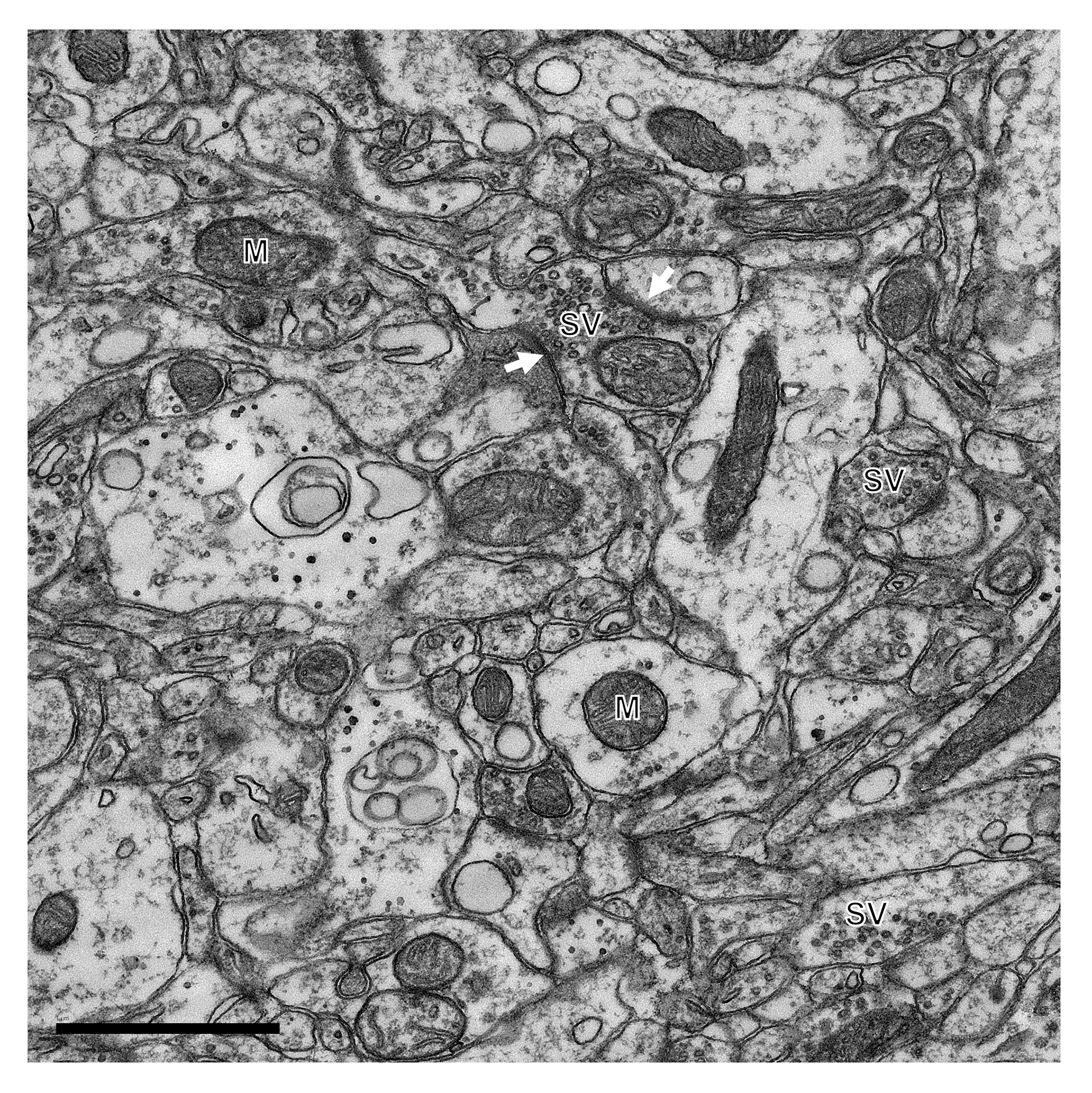

Supplement: Supplementary Figure 5 — TEM image of mouse cortex after fixation and staining with PLT-LTS with heavy metal enhancement. Synapses (arrow), synaptic vesicles (SV) and mitochondria (M) are well preserved. Scale bar 1 μm. [file Image_5.TIF]

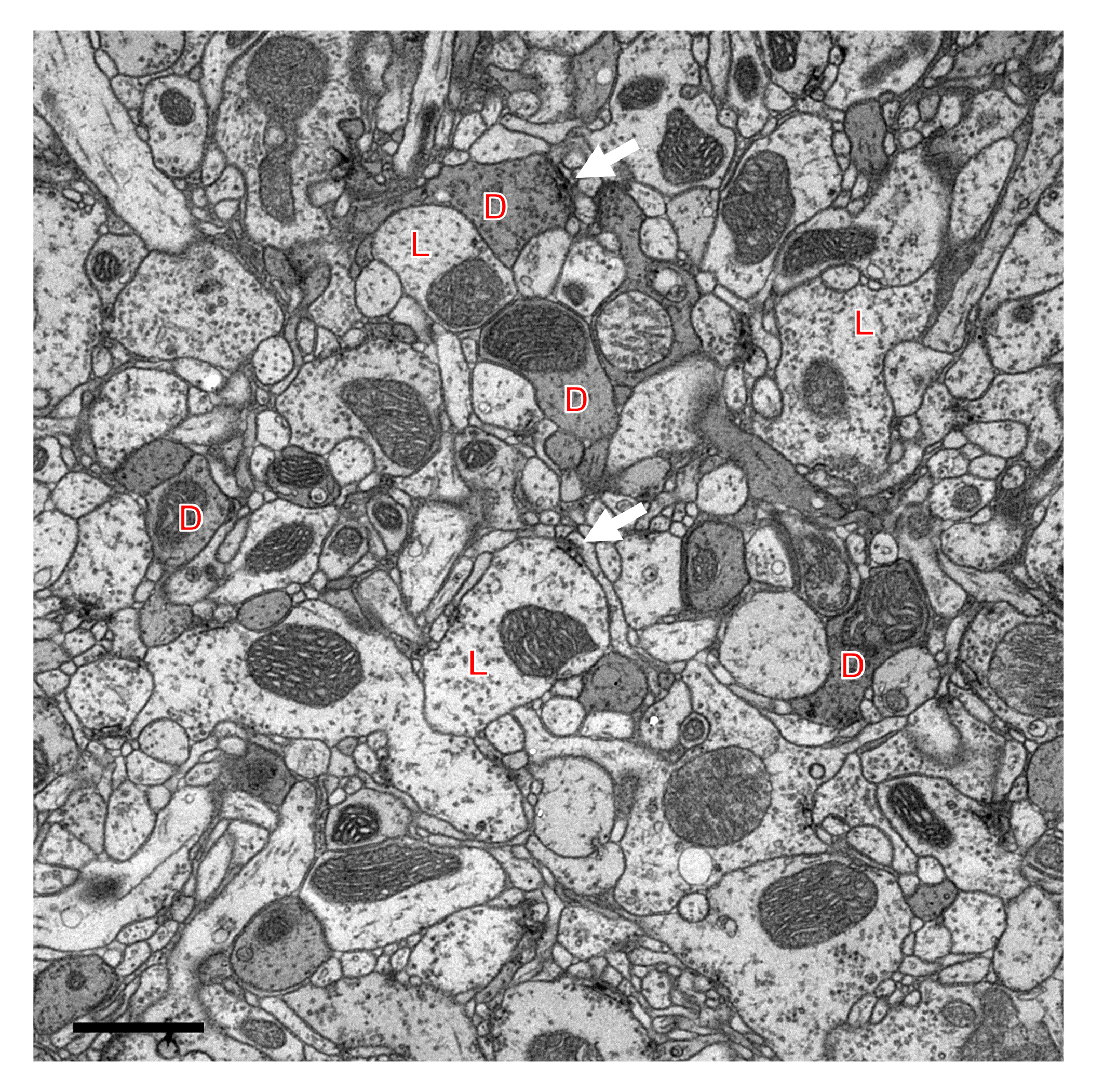

Supplement: Supplementary Figure 6 — TEM image of Drosophila optic lobe after fixation and staining with PLT-LTS. Most neuronal processes have light (L) cytoplasm, but some have dark (D). Synapses (arrow) are detectable in light and dark processes in this sample. Scale bar 1 μm. [file Image_6.TIF]
