# Supplementary material for: En bloc preparation of Drosophila brains enables high-throughput FIB-SEM connectomics
Source: Front Neural Circuits. 2022 Dec 16;16:917251. doi: 10.3389/fncir.2022.917251 (PMC9801301; doi:10.3389/fncir.2022.917251)
Supplement: Supplementary file 8 [file Table_1.DOCX]

**Supplementary Table 1: Timetable of temperature and solvent changes during PLT-LTS processing.**

| 22°C | 1°C | 1°C to -5°C | -5°C to -10°C | -10°C to -15°C | -15°C to -20°C | -20°C to -25°C | -25°C |  |
| --- | --- | --- | --- | --- | --- | --- | --- | --- |
| 2 hrs | 2.5%GA +2.5%PFA |  |  |  |  |  |  |  |
| 30 min |  | 0.5%O_s_O_4_ |  |  |  |  |  |  |
| 30 min |  | 0.5%UA |  |  |  |  |  |  |
| 20 min |  | 0.8% O_s_O_4_ |  |  |  |  |  |  |
| 36 min |  |  | 10% acetone |  |  |  |  |  |
| 30 min |  |  |  | 30% acetone |  |  |  |  |
| 30 min |  |  |  |  | 50% acetone |  |  |  |
| 30 min |  |  |  |  |  | 70% acetone |  |  |
| 30 min |  |  |  |  |  |  | 80% acetone |  |
| 30 min |  |  |  |  |  |  |  | 90% acetone |
| 30 hrs |  |  |  |  |  |  |  | 1% O_s_O_4_ + 0.2% UA in 97% acetone |
| 5 hrs |  | Temperature raising up from 25°C to 0°C, samples in 1% O_s_O_4_ + 0.2% UA in 97% acetone | | | | | | |
| 40 min | 100% acetone | |  |  |  |  |  |  |
